# 20-year trends in prevalence of overweight and obesity among children aged 0-6 in Harbin, China: A multiple cross-sectional study

**DOI:** 10.1371/journal.pone.0198032

**Published:** 2018-06-04

**Authors:** Wei Liu, Qin Li, Hui Li, Jia Li, Hai-Jun Wang, Bin Li

**Affiliations:** 1 Department of Social Medicine, School of Public Health, Harbin Medical University, Harbin, China; 2 Department of Maternal and Child Health, School of Public Health, Peking University, Beijing, China; 3 Department of Growth and Development, Capital Institute of Pediatrics, Beijing, China; 4 Harbin Maternal and Child Health Care Hospital, Harbin, China; The Ohio State University, UNITED STATES

## Abstract

To examine the 20-year trends in the combined prevalence of overweight and obesity among children aged 0–6 years between 1995 and 2015 in Harbin, China, we selected altogether 49,553 children aged 0–6 years old by using a multistage stratified cluster sampling methods in Harbin, one provincial capital city in northeast China in 1995, 2005 and 2015. Height and weight information of the children were collected. We used the child growth standards of World Health Organization to calculate the Z-scores for body mass index (BMI). Cut-offs recommended by World Health Organization and International Obesity Task Force were used to define overweight and obesity for each children. We found there is no difference between boys’ BMI and girls’ among newborns in each survey point (p>0.05), but in older age groups, the BMI of boys was higher than that of girls (p<0.05). From 1995 to 2015, the average BMI was increasing continuously among boys older than 42 months and girls older than 48 months (*p*<0.01 for linear trend across year) in Harbin. The combined prevalence of overweight and obesity increased from 2.6% in 1995 to 7.6% in 2015. For every 10-year the risk of combined overweight and obesity in children aged 0–6 years increased by 167% (95%CI: 146%, 188%, p<0.01). The combined prevalence of overweight and obesity in most age subgroups showed an increasing trend over time (*p*<0.01 for trend test across survey year). The age when the combined prevalence of overweight and obesity dramatically increased was earlier in 2015 than that in 2005 and 1995. In conclusion, there was an increasing trend of the combined prevalence of overweight and obesity during the past 20 years in Harbin and the age when the prevalence dramatically increased became earlier. Comprehensive intervention should be undertaken among younger children to prevent and control children’s overweight and obesity.

## Introduction

Over the past 40 years, the obesity prevalence increased from 3.2% in 1975 to 10.8% in 2014 in men, and from 6.4% to 14.9% in women worldwide [1]. A previous study predicted that the global obesity prevalence will reach 18% in male and surpass 21% in female by 2025 under the current growth trend [1]. One of the main disconcerting aspects of the global obesity epidemiology is the fast-growing combined prevalence of childhood overweight and obesity. Overweight and obesity in the early life are highly associated with not only short-term health outcomes but also long-term adverse effects. Many Cross-sectional studies indicated overweight and obesity children presented abnormal lipids, insulin and blood pressure level compared with normal weight children.[2–4] Several cohort studies reported overweight and obesity during childhood was associated with increased overall mortality, and specifically with increased risk of cardiovascular disease and diabetes in adults [2–4]. On the other hand, studies also indicated that the early development of overweight in childhood was also related to subsequent psychosocial difficulties in child and adolescent, such as depression and poor academic performance [2]. In conclusion, childhood overweight and obesity could result in serious health consequences and social economic burden.

With the rapid economic development and obvious transition of lifestyle in last two decades, the combined prevalence of overweight and obesity among children dramatically increased in some developing countries [5–7]. Studies from The Chinese National Survey on Students’ Constitution and Health (CNSSCH), the largest nationally representative sample of school-age children in China, reported that the prevalence of obesity dramatically increased from 0.2% in 1985 to 8.1% in 2010 among school-age children of China [6]. Another study from Mexico also reported that the prevalence of obesity increased from 8.9% in 1999 to 14.6% in 2012 among school-age children [7]. With the increasing prevalence of obesity among school-age children, evaluating the long-term epidemiological transitions of overweight and obesity in the earlier childhood to develop intervention policy is urgent, especially in developing countries such as China.

However, most previous studies regarding the long-term trend of overweight and obesity epidemic among Chinese children focused on school-age children (aged 7–18 years) [5, 6, 8–10]. As far as we know, only a few studies reported the epidemiological transitions of obesity among Chinese children aged 0–6 years. Zong et al. reported that the prevalence of obesity (defined by National Centre for Health Statistics/World Health Organization reference) increased from 0.9% in 1986 to 3.4% in 2006 among children aged 0–6 years in nine cities of China [8]. Xiao et al. reported that the prevalence of obesity (defined by World Health Organization child growth standards) increased from 8.8% in 2006 to 10.1% in 2014 among children aged 5–6 years in Tianjin, China [11]. However, there is still a gap in the evidence regarding the long-term trend in the combined prevalence of overweight and obesity in children aged 0–6 years during the last two decades. No study regarding the trend in children’s overweight and obesity has been conducted in Harbin, one provincial capital city of Heilongjiang province in northeast China. The objective of the present study was to examine the 20-year trends in the combined prevalence of overweight and obesity among children aged 0–6 years between 1995 and 2015 in Harbin, China.

## Method

### Ethics approval

Members of the survey’s staff explained to the parents of children the purpose of the survey and ensured that each participants has given a verbal agreement. The Project was approved by the Ethics Committee of the Capital Institute of Pediatrics (SHERLL 2015009). All data were fully anonymized before we accessed them.

### Participants

The National Survey on the Physical Growth and Development of Children in the Nine Cities of China (NSPGDC) was conducted across nine cities of China every 10 years, which involved the largest sample of pre-school child in China. In NSPGDC, newborns were selected from hospitals, children aged 1 month to 3 years were selected from communities, and children over 3 years (including 3 years) were selected from kindergartens through multistage stratified cluster sampling. Briefly, survey area (including both urban and rural areas) of each city was decided based on the social economic level. Hospitals, communities and kindergartens were selected randomly as cluster from all the hospitals, communities and kindergartens in each survey areas, and then all the children in the selected clusters were included in the survey after having informed consent. The clusters were fixed across three survey years. Detailed information of NSPGDC has been described elsewhere [12, 13]. In the present cross-sectional study, we collected data of the children aged 0–6 years from Harbin city (covering an area of 53523.5 km^2^, with 10.6 million residents in 2015) in the database of 1995, 2005, 2015 cycles of NSPGDC. All participants of the present study had medical examination before measurement, to ensure that they had no physical and mental disorders, including respiratory and cardiovascular disorders. We finally included 49,553 children aged 0–6 years from Harbin city in this study. The sample size ranged from 241 to 461 in each gender- and age-specific subgroup among children aged 0–6 years. (See S1 Table for additional information about sample size).

### Measures

Weight of newborns was measured by newborn scale after putting them on the scale and recorded in grams to the nearest 10 g. Weight of children aged 1 month to 6 years was measured by their parents holding them or sitting/standing alone in the middle of the electronic scale and recorded in kilograms to the nearest 0.01 kg. Length of children under 3 years of age and height of children aged 3 years or older were measured by using infant meter and stadiometer, respectively and then recorded to the nearest to 0.1 cm. Subjects were required to wear only light clothes, barefoot and at ease when being measured. Both the scales and stadiometers were calibrated before use. All measurements were conducted from July to November in each survey year by a team of health professionals in each survey site, who were required to pass a training course for the measurements. The detailed information of the measurements had been published elsewhere [6, 13]. Body mass index (BMI, kg/m^2^) was calculated as weight (kilograms) divided by height/length (m) squared. We used the 2007 World Health Organization (WHO) Growth Chart to calculate Z-score for BMI and then defined the nutritional status for children aged 0–24 months [14]. As recommended by WHO Growth Chart, the children with BMI Z-score greater than 2SD were defined as overweight and those BMI Z-score greater than 3SD were defined as obesity. For children older than 24 months, we use the definitions from the International Obesity Task Force (IOTF) to define overweight and obesity [15]. We also use the overweight and obesity definitions of China which derives from National Survey on the Physical Growth and Development of Children in the nine cities of China as sensitivity analysis [16].

### Statistical analyses

We used the R software, version 3.4.2 (R Core Team) to analyse the data. Mean and standard deviation of BMI and the combined prevalence of overweight and obesity among each subgroup was calculated and reported. Differences in the mean values of BMI between girls and boys were tested by analysis of variance (ANOVA). To test trends across years, we regressed survey years as an ordinal variable for the continuous outcome of BMI with a linear regression model. Differences in the combined prevalence of overweight and obesity between girls and boys were tested by Chi-square tests. To test trends across years, we also regressed survey years as an ordinal variable for the binary outcome of combined overweight and obesity with a logistic regression model. A value of *p* < 0.05 from two-side test was considered statistically significant.

## Results

A total of 49,553 children aged 0–6 years were collected from 1995 to 2015. It included 24,777 boys (50%) and 24,776 girls (50%). The total number of children evaluated from 17,533 in 1995 to 18,625 in 2015. The mean values of weight, height/length from 1995 to 2015 were showed S1 and S2 Figs. The mean weight, height/length of children aged 0–6 years increased from 1995 to 2005 in most age-specific subgroup.

**Fig 1:**
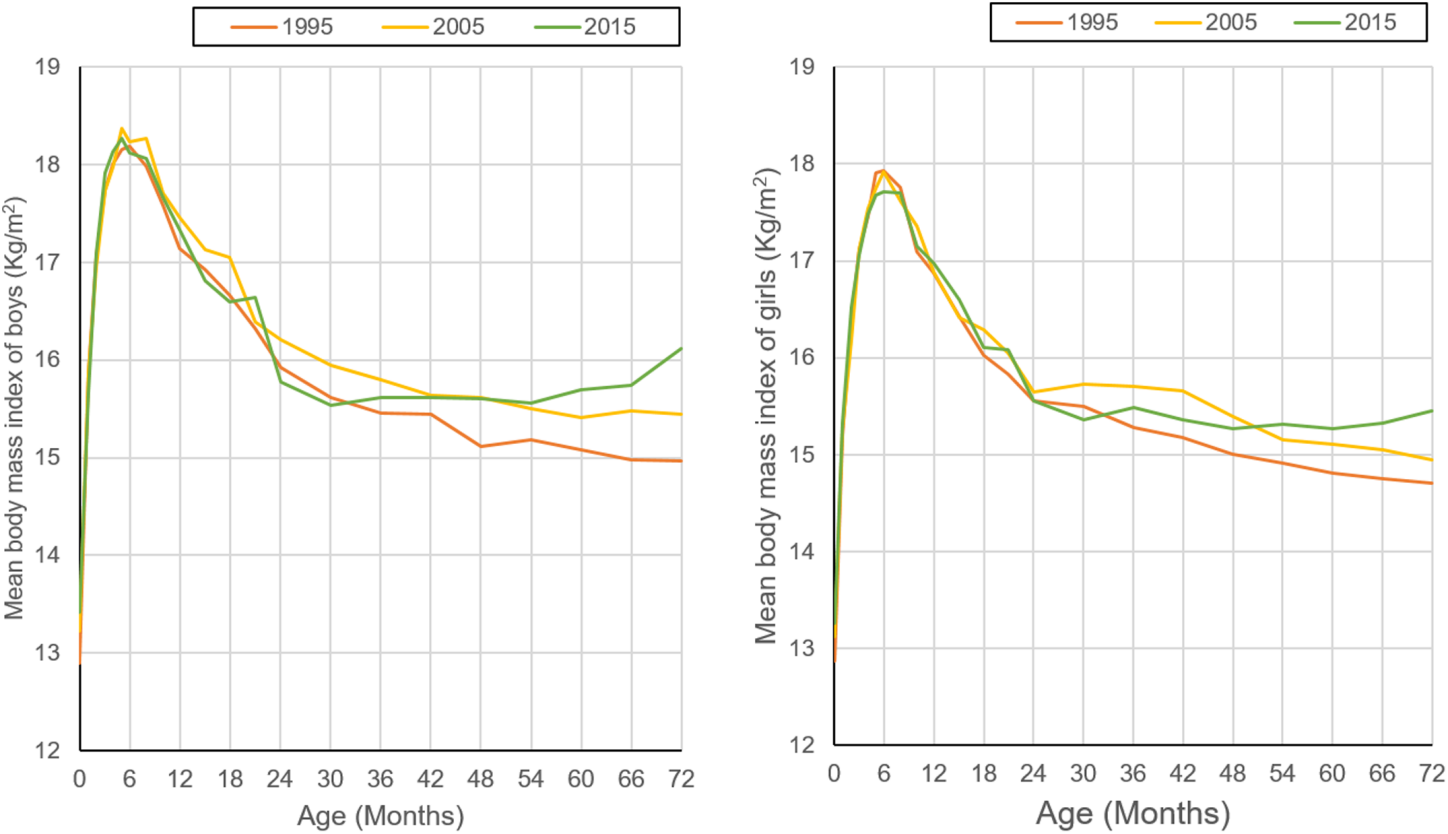
20-year changes of body mass index (BMI) in boys and girls aged 0–72 months in Harbin.

Fig 1 and Table 1 shows the mean values of children’s BMI for each age-specific subgroup and the curve of BMI-for-age of children aged 0–6 years in each survey year. There is no difference between boys’ BMI and girls’ among newborns in each survey point (p>0.05), but in most age groups, the BMI of boys was higher than that of girls (p<0.05). The shape of the curve among children younger than 10 months was steady during the past 20 years (*p*>0.05 for trend test across survey year). But we found the mean BMI of children was increasing continuously over the past 20 years among boys older than 42 months and girls older than 48 months (*p*<0.01 for trend test across survey year).

**Table 1:** Body mass index of children of 1995, 2005 and 2015 survey.

Age (Months)	1995				2005				2015				*p* value for trend ^a^		
	All (Mean±SD)	Boys (Mean±SD)	Girls (Mean±SD)	*p* value ^b^	All (Mean±SD)	Boys (Mean±SD)	Girls (Mean±SD)	*p* value ^b^	All (Mean±SD)	Boys (Mean±SD)	Girls (Mean±SD)	*p* value ^b^	All	Boys	Girls
**0–3 Days**	12.88±1.14	12.89±1.18	12.87±1.10	0.77	13.17±1.27	13.22±1.15	13.12±1.38	0.32	13.34±1.14	13.42±1.09	13.26±1.18	0.10	<0.01	<0.01	<0.01
**1~**	15.53±1.25	15.87±1.19	15.19±1.23	<0.01	15.57±1.45	15.87±1.51	15.26±1.33	<0.01	15.49±1.37	15.66±1.35	15.32±1.38	<0.01	0.57	0.03	0.17
**2~**	16.73±1.34	17.06±1.39	16.39±1.19	<0.01	16.56±1.43	16.95±1.41	16.17±1.35	<0.01	16.80±1.61	17.09±1.54	16.52±1.63	<0.01	0.32	0.81	0.20
**3~**	17.44±1.46	17.75±1.51	17.12±1.33	<0.01	17.42±1.47	17.73±1.41	17.12±1.47	<0.01	17.48±1.66	17.92±1.66	17.06±1.54	<0.01	0.57	0.12	0.55
**4~**	17.74±1.24	18.01±1.20	17.46±1.22	<0.01	17.75±1.43	17.97±1.45	17.54±1.37	<0.01	17.80±1.72	18.13±1.74	17.48±1.64	<0.01	0.37	0.27	0.82
**5~**	18.03±1.21	18.15±1.18	17.91±1.23	0.01	18.06±1.54	18.37±1.67	17.75±1.32	<0.01	17.97±1.71	18.27±1.68	17.68±1.69	<0.01	0.44	0.25	0.02
**6~**	18.06±1.30	18.19±1.32	17.93±1.26	0.01	18.08±1.58	18.24±1.58	17.92±1.56	0.01	17.92±1.58	18.12±1.67	17.71±1.46	<0.01	0.05	0.54	0.02
**8~**	17.87±1.32	17.98±1.30	17.76±1.34	0.02	17.94±1.63	18.27±1.69	17.62±1.50	<0.01	17.88±1.66	18.06±1.62	17.70±1.68	<0.01	0.92	0.48	0.59
**10~**	17.34±1.37	17.58±1.30	17.09±1.40	<0.01	17.54±1.39	17.71±1.39	17.36±1.38	<0.01	17.41±1.51	17.67±1.46	17.15±1.52	<0.01	0.36	0.37	0.61
**12~**	17.00±1.27	17.14±1.22	16.87±1.31	<0.01	17.18±1.60	17.46±1.55	16.89±1.60	<0.01	17.15±1.49	17.34±1.49	16.98±1.47	<0.01	0.03	0.05	0.26
**15~**	16.67±1.18	16.92±1.12	16.43±1.19	<0.01	16.77±1.45	17.13±1.48	16.41±1.34	<0.01	16.71±1.54	16.81±1.47	16.60±1.61	0.04	0.64	0.25	0.07
**18~**	16.34±1.24	16.66±1.28	16.02±1.10	<0.01	16.66±1.43	17.05±1.48	16.29±1.28	<0.01	16.36±1.52	16.60±1.52	16.11±1.48	<0.01	0.88	0.48	0.35
**21~**	16.08±1.11	16.32±1.12	15.83±1.04	<0.01	16.22±1.43	16.39±1.44	16.05±1.40	<0.01	16.36±1.50	16.64±1.55	16.08±1.39	<0.01	<0.01	0.00	0.01
**24~**	15.74±1.08	15.92±1.06	15.55±1.07	<0.01	15.93±1.30	16.21±1.29	15.65±1.26	<0.01	15.66±1.34	15.77±1.29	15.56±1.39	0.02	0.18	0.05	0.95
**30~**	15.55±1.07	15.61±1.07	15.50±1.07	0.15	15.84±1.20	15.95±1.12	15.73±1.27	0.02	15.45±1.26	15.54±1.25	15.36±1.26	0.03	0.05	0.35	0.07
**36~**	15.37±1.10	15.46±1.14	15.28±1.05	0.02	15.75±1.21	15.80±1.24	15.70±1.17	0.31	15.55±1.28	15.61±1.25	15.48±1.30	0.13	<0.01	0.08	0.05
**42~**	15.31±1.05	15.44±1.08	15.18±1.01	<0.01	15.65±1.31	15.64±1.30	15.66±1.33	0.83	15.49±1.33	15.62±1.31	15.36±1.34	<0.01	<0.01	0.03	0.06
**48~**	15.06±1.09	15.12±1.09	15.00±1.09	0.12	15.50±1.35	15.62±1.33	15.39±1.36	0.04	15.43±1.42	15.60±1.43	15.27±1.40	<0.01	<0.01	<0.01	<0.01
**54~**	15.05±1.07	15.18±1.13	14.91±0.99	<0.01	15.33±1.32	15.50±1.34	15.15±1.28	<0.01	15.43±1.52	15.56±1.58	15.31±1.46	0.02	<0.01	<0.01	<0.01
**60~**	14.94±1.14	15.08±1.16	14.81±1.11	<0.01	15.26±1.35	15.41±1.43	15.11±1.24	0.01	15.48±1.75	15.69±1.87	15.27±1.61	<0.01	<0.01	<0.01	<0.01
**66~**	14.86±1.12	14.98±1.11	14.75±1.11	<0.01	15.27±1.41	15.48±1.50	15.05±1.28	<0.01	15.54±1.82	15.74±1.83	15.32±1.78	<0.01	<0.01	<0.01	<0.01
**72–84**^c^	14.83±1.19	14.97±1.16	14.70±1.20	<0.01	15.20±1.50	15.45±1.57	14.95±1.39	<0.01	15.78±2.00	16.12±2.08	15.45±1.86	<0.01	<0.01	<0.01	<0.01

^a^. *p* value for trend derived from linear regression model;

^b^. *p* value for differences between genders;

^c^. 84 months is not included.

**Fig 2:**
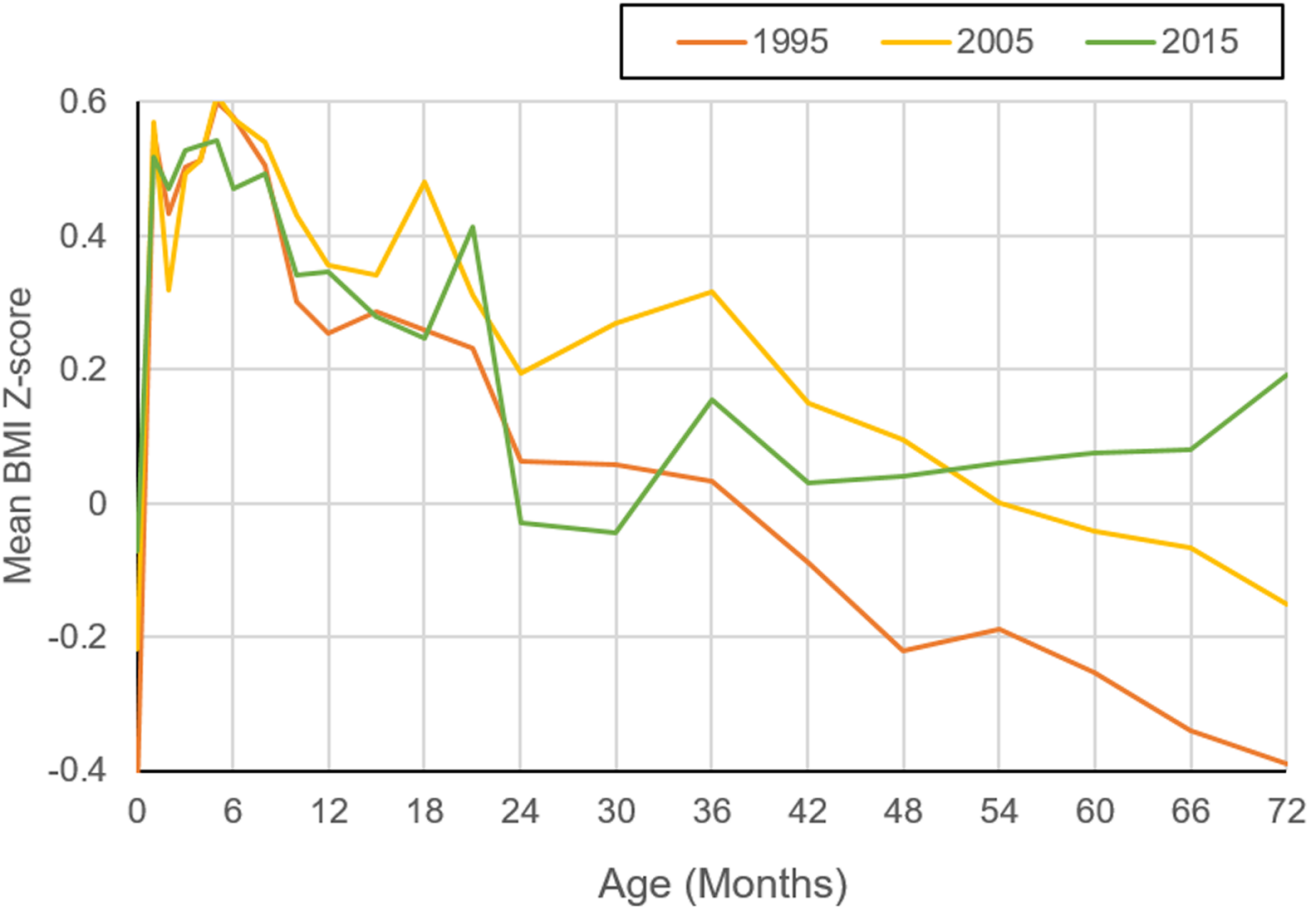
20-year changes of body mass index (BMI) z-score in children aged 0–72 months in Harbin.

Fig 2 shows the curve of BMI Z-score for age of children aged 0–6 years in each survey year. We found BMI Z-score of children older than 42 months were increasing continuously in 2015, which was related to the timing of adiposity rebound. However, we can’t identify when the increase in BMI Z-score took place after the nadir among the children aged 0–6 years in 2005 and 1995.

**Fig 3:**
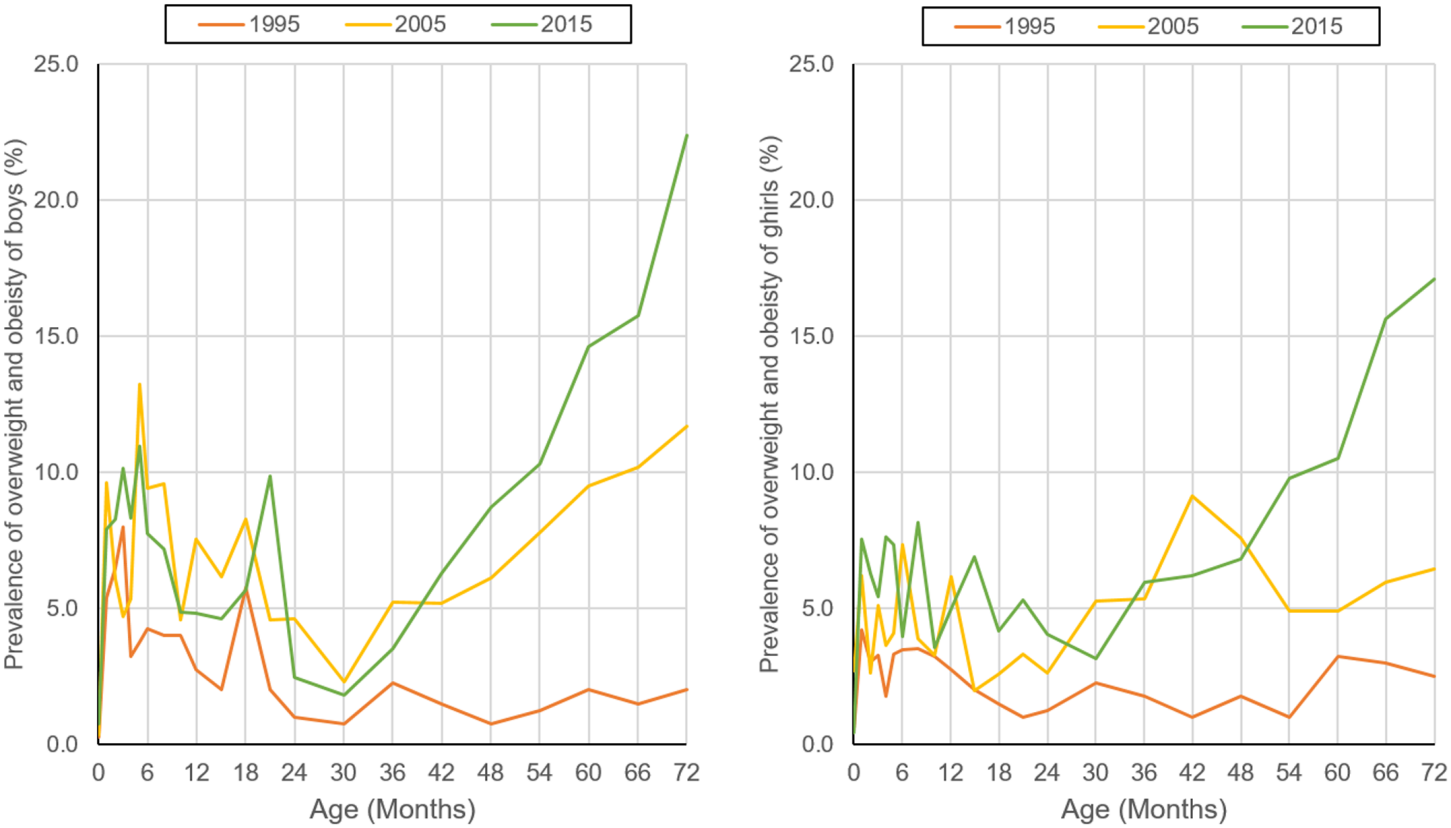
20-year changes of the combined prevenance of overweight and obesity in boys and girls aged 0–72 months in Harbin.

Table 2 shows the combined prevalence of overweight and obesity in each age group of each survey year. The combined prevalence of overweight and obesity increased from 2.6% in 1995 to 7.6% in 2015 in children aged 0–6 years in Harbin. For every 10-year the risk of combined overweight and obesity in children aged 0–6 years increased by 167% (95%CI: 146%, 188%, p<0.01). In addition, the combined prevalence of overweight and obesity in most age subgroups showed an increasing trend over time (*p*<0.01 for trend test across survey year). The combined prevalence of overweight and obesity was higher among boys than girls at each survey point (*p*<0.01). Fig 3 shows the curve of the age specific combined prevalence of overweight and obesity. We found the prevalence increased with age continually in boys older than 42 months in 2010 and 30 months in 2015. The prevalence increased continually with age in girls older than 30 months in 2015. It indicated that the age when the combined prevalence of overweight and obesity dramatically increased was earlier in 2015 than that in 2005 and 1995. When we use the overweight and obesity definitions of China, the results do not change (S3 Fig).

**Table 2:** The combined prevalence of overweight and obesity by age group of 1995, 2005 and 2015 survey.

Age	1995				2005				2015				*p* value for trend ^b^
	All (%)	Boys (%)	Girls (%)	*p* value ^a^	All (%)	Boys (%)	Girls (%)	*p* value ^a^	All (%)	Boys (%)	Girls (%)	*p* value ^a^	
**0–3 Days**	0.4	0.3	0.5	0.99	1.5	0.3	2.7	0.04	0.6	0.8	0.4	1.00	0.49
**1~**	4.8	5.4	4.2	0.55	7.9	9.6	6.2	0.16	7.7	7.9	7.5	0.94	0.02
**2~**	4.8	6.5	3.0	0.03	4.3	6.1	2.6	0.06	7.3	8.3	6.3	0.34	0.03
**3~**	5.6	8.0	3.3	<0.01	4.9	4.7	5.1	0.97	7.7	10.1	5.4	0.02	0.09
**4~**	2.5	3.3	1.8	0.26	4.5	5.4	3.6	0.41	8.0	8.3	7.6	0.82	<0.01
**5~**	3.5	3.8	3.3	0.88	8.7	13.3	4.1	<0.01	9.1	11.0	7.4	0.11	<0.01
**6~**	3.9	4.3	3.5	0.71	8.4	9.4	7.4	0.44	5.9	7.8	4.0	0.02	0.09
**8~**	3.8	4.0	3.5	0.85	6.7	9.6	3.9	0.01	7.7	7.2	8.2	0.67	<0.01
**10~**	3.6	4.0	3.3	0.71	3.9	4.6	3.3	0.53	4.2	4.8	3.6	0.44	0.55
**12~**	2.8	2.8	2.8	1.00	6.9	7.5	6.2	0.61	4.9	4.8	5.0	1.00	0.05
**15~**	2.0	2.0	2.0	1.00	4.1	6.2	2.0	0.01	5.8	4.6	6.9	0.19	<0.01
**18~**	3.6	5.8	1.5	<0.01	5.4	8.3	2.6	<0.01	4.9	5.7	4.2	0.39	0.21
**21~**	1.5	2.0	1.0	0.38	4.0	4.6	3.3	0.55	7.6	9.9	5.3	0.02	<0.01
**24~**	1.1	1.0	1.3	1.00	3.6	4.6	2.6	0.26	3.3	2.5	4.1	0.25	0.01
**30~**	1.5	0.8	2.3	0.15	3.8	2.3	5.3	0.09	2.5	1.8	3.1	0.28	0.23
**36~**	2.0	2.3	1.8	0.80	5.3	5.2	5.4	1.00	4.7	3.5	5.9	0.13	0.01
**42~**	1.3	1.5	1.0	0.74	7.2	5.2	9.1	0.08	6.3	6.3	6.2	1.00	<0.01
**48~**	1.3	0.8	1.8	0.22	6.8	6.1	7.6	0.59	7.8	8.7	6.8	0.35	<0.01
**54~**	1.1	1.3	1.0	1.00	6.3	7.8	4.9	0.20	10.0	10.3	9.8	0.88	<0.01
**60~**	2.6	2.0	3.3	0.38	7.2	9.5	4.9	0.04	12.6	14.6	10.5	0.08	<0.01
**66~**	2.3	1.5	3.0	0.23	8.1	10.2	5.9	0.07	15.7	15.8	15.6	1.00	<0.01
**72–84**^c^	2.3	2.0	2.5	0.81	9.1	11.7	6.4	0.03	19.7	22.4	17.1	0.06	<0.01
**All**	2.6	3.0	2.3	0.01	5.8	6.9	4.8	<0.01	7.6	8.1	7.0	<0.01	<0.01

^a^. *p* value for differences between genders.

^b^. *p* value for trend derived from logistic regression model.

^c^. 84 months is not included.

## Discussion

In the panel study over the last two decades, we found the average BMI was increasing continuously among boys older than 42 months and girls older than 48 months in Harbin, one provincial capital city of Heilongjiang province in northeast China. We also found that the combined prevalence of overweight and obesity among children aged 0–6 years showed an increasing trend over time. In addition, we found the age when the combined prevalence of overweight and obesity dramatically increased was earlier in 2015 than that in 2005 and 1995. Facing the increasing trend of the combined prevalence of overweight and obesity among younger children and the earlier outbreak ages of overweight and obesity, it is necessary to take energetic interventions during early childhood.

We found two previous studies also observed that there is a growth trend in the prevalence of overweight or obesity among younger children with various age subgroups during the past two decades. Xiao et al. collected the annul health exam data of 145,078 children aged 3–6 years from 46 kindergartens during 2006 to 2014 and reported that the prevalence of obesity (defined as BMI z-scores >2SD) increased significantly from 8.8% in 2006 to 10.1% in 2010 and then kept stable until 2014 among children aged 5–6 years in Tianjin, an eastern city of China. However, they didn’t found a significantly increasing trend of both overweight (defined as BMI z-scores >2SD) and obesity (defined as BMI z-scores >3SD) among children aged 3–4 years [11]. Chen et al. collected the anthropometry data of 71,229 children aged 0–5 years at three time points: 2009, 2012 and 2015 in Xiamen, a southeast city of China, and reported that the prevalence of obesity (defined as BMI for age ≥ P95) increased significantly from 0.9% in 2009 to 1.5% in 2015 among boys and from 0.8% to 1.3% among girls. They also reported that the prevalence of overweight (defined as BMI for age ≥ P85) increased significantly from 1.5% in 2009 to 6.5% in 2015 among boys and from 1.0% to 6.1% among girls [17].

We noticed that the previous study reported the prevalence of obesity among pre-school children became stabilized in the eastern city of China recently [11], which may be due to the increasing awareness of the risk of childhood obesity in the families, schools and society and more obesity intervention programs in recent years. Studies from Sweden [18] or Australia [19] also reposted no recent increase in the prevalence of childhood obesity. In France [20] and Greece [21], studies reported that the prevalence of obesity in children even fell in recent years. However, our results showed a continually increasing trend of combined overweight and obesity in children aged 0–6 years in Harbin, a northeast city of China. A study from a southern city of China (Xiamen) also showed an increasing trend of combined overweight and obesity in children aged 0–5 years [17]. It indicated there are regional disparities in the trends in combined prevalence of overweight and obesity, so it is necessary to take interventions during early childhood, in order to control the increasing trend of combined overweight and obesity in adolescents and adults in the areas with similar increasing trend.

In addition, in the present study we found the onset ages of combined overweight and obesity of both boys and girls were earlier in 2015 than that in 2005 and 1995. The shift might be related to the increasing combined prevalence of overweight and obesity among school-age children. We also found BMI Z-score of children older than 42 months were increasing continuously in 2015, which was related to the timing of adiposity rebound, but we can’t identify when the increase in BMI Z-score took place after the nadir in 1995 and 2005. It indicated the timing of adiposity rebound became earlier in 2015 than before. Previous Studies in Poland and Czech also reported that the adiposity rebound age became earlier during the last three decades. [22, 23] Studies from China also reported an earlier puberty onset age, which related to adiposity rebound, during the last three decades. [24] A number of studies have suggested that earlier adiposity rebound increases obesity risk [22], which could be related to the increasing trend of combined overweight and obesity among school-age children. Children who became overweight or obesity in their earlier life are highly associated with metabolic (e.g., diabetes and metabolic syndrome), orthopaedic (e.g., slipped capital femoral epiphysis and Blount’s disease), cardiovascular (e.g., hypertension and atherosclerosis), psychological (e.g., depression and poor quality of life), neurological (e.g., pseudotumor cerebri), hepatic (e.g., non-alcoholic fatty liver) pulmonary (e.g., obstructive sleep apnoea) and renal (e.g., proteinuria) disorders [2, 25], which can hardly be reversed and may result in heavier public health burden of metabolic diseases in the future. These findings indicated that it is more important to take effective interventions among younger children to reverse the increasing trend of combined overweight and obesity in these areas. As children younger than 3 years usually stay with their caregivers, promoting proper health education program among the caregivers of children younger than 3 years in the community might be helpful [26].

Many studies showed that dietary and exercise habit are highly related to early childhood overweight or obesity [4]. With the rapid economic development from 1980s, China is suffering from epidemiological and demographic transitions [27]. Traditional diets and activity behaviours have been progressively replaced by high energy intake and sedentary lifestyles, which contribute to increase of childhood obesity prevalence [28, 29]. Previous studies indicated that dietary and exercise habit are being established and can be more shapable during pre-school years than in later childhood [30, 31]. Our study found younger children also showed the increasing combined prevalence of overweight and obesity, and its onset age became earlier. It means developing effective intervention such as lifestyle improvement, food selection, nutritional supplement and physical activity promotion for children aged 0–6 years to help them improve their dietary and exercise habit might be an important step for combating the childhood obesity epidemic. For example, as caregivers play an important role in helping young children learn and practice, healthful behaviour such as increasing fruits and vegetables consumption (“5 servings per day”), switching from full-fat to less-fat or fat-free food after 2 years of age, preparing and eating family meals at home, avoiding sugared beverages, increasing daily physical activity (“1 hour per day”), and limiting screen time (“<2 hour per day”) should be transferred to caregivers and then help them to guide their children’s behaviours. In addition, we noticed policies such as banned stores which sold high fat food near primary school have been implemented in some cities of China, but financial policies such as tax on beverage and high fat food could also been improved, which may help to control the epidemic of overweight and obesity.

There are some limitations in this study. First, as data for this study were obtained from three independent cross-sectional surveys rather than from a fixed cohort, we could not investigate the trend in the combined prevalence of overweight and obesity longitudinally. Second, the detailed information of individual socioeconomic status, behaviors, parents’ knowledge and attitude were not available, which limited us to determine the reasons for the trend in the combined prevalence of overweight and obesity among children aged 0–6 years. There are several strengths in this study. First of all, this study was based on the large panel data of children in Harbin over 20 years. The data of children’s weight and height measured by a team of trained health professionals. In addition, due to the similar sample size in different age-specific subgroups, we can estimate the change of the onset age of combined overweight and obesity during the past 20 years. We found the timing of adiposity rebound become earlier in 2015 than before, which could be a reason for the trend. Second, this study was conducted in pre-school children of Harbin, one provincial capital city of Heilongjiang province in northeast China, which has no study reporting the trends in nutrition status of pre-school children. It is the first study which reported the trend in the combined overweight and obesity prevalence among children aged 0–6 years in northeast China during the recent two decades.

## Conclusion

We found that the combined prevalence of overweight and obesity among children aged 0–6 years had an increasing trend during the past 20 years. The age when the combined prevalence of overweight and obesity dramatically increased was the earlier in 2015 than that in 2005 and 1995. Comprehensive intervention should be undertaken among younger children to prevent and control children’s overweight and obesity by implementing regular monitoring, nutrition education, aerobic exercise, and healthy eating behaviours.

## Supporting information

S1 Table Sample sizes of children aged 0–6 years in Harbin. * 84 months is not included.
(DOCX)

S1 Fig Weights of boys and girls aged 0–6 years in Harbin of 1995, 2005 and 2015 cycles of survey. (TIF)

S2 Fig Heights/Length of boys and girls aged 0–6 years in Harbin of 1995, 2005 and 2015 cycles of survey. (TIF)

S3 Fig 20-year changes of prevalence of overweight/obesity in boys and girls aged 0–72 months in Harbin (Using the definition of overweight/obesity from China). (TIF)
