# Supplementary material for: 20-year trends in prevalence of overweight and obesity among children aged 0-6 in Harbin, China: A multiple cross-sectional study
Source: PLoS One. 2018 Jun 4;13(6):e0198032. doi: 10.1371/journal.pone.0198032 (PMC5986120; doi:10.1371/journal.pone.0198032)
Supplement: S1 Table — * 84 months is not included. (DOCX) [file pone.0198032.s001.docx]

**S1 Table. Sample Sizes of children aged 0-6 years in Harbin.**

|  | | | | | | | | |
| --- | --- | --- | --- | --- | --- | --- | --- | --- |
| **Age**  **(Months)** | **1995** | |  | **2005** | |  | **2015** | |
|  | **Boys** | **Girls** |  | **Boys** | **Girls** |  | **Boys** | **Girls** |
| **0-3 Days** | 387 | 384 |  | 296 | 297 |  | 262 | 241 |
| **1~** | 391 | 381 |  | 302 | 307 |  | 404 | 385 |
| **2~** | 400 | 395 |  | 297 | 304 |  | 399 | 399 |
| **3~** | 400 | 398 |  | 297 | 293 |  | 385 | 406 |
| **4~** | 400 | 400 |  | 299 | 303 |  | 397 | 407 |
| **5~** | 400 | 394 |  | 302 | 295 |  | 383 | 394 |
| **6~** | 400 | 401 |  | 308 | 299 |  | 438 | 430 |
| **8~** | 400 | 400 |  | 303 | 309 |  | 446 | 454 |
| **10~** | 400 | 400 |  | 306 | 306 |  | 434 | 449 |
| **12~** | 400 | 400 |  | 305 | 308 |  | 437 | 442 |
| **15~** | 400 | 400 |  | 308 | 306 |  | 434 | 434 |
| **18~** | 400 | 400 |  | 302 | 309 |  | 441 | 431 |
| **21~** | 400 | 400 |  | 306 | 302 |  | 436 | 432 |
| **24~** | 400 | 400 |  | 303 | 307 |  | 446 | 444 |
| **30~** | 400 | 400 |  | 304 | 305 |  | 448 | 446 |
| **36~** | 400 | 401 |  | 307 | 317 |  | 426 | 421 |
| **42~** | 400 | 401 |  | 308 | 307 |  | 461 | 451 |
| **48~** | 400 | 400 |  | 310 | 304 |  | 447 | 440 |
| **54~** | 400 | 400 |  | 309 | 306 |  | 446 | 450 |
| **60~** | 400 | 400 |  | 306 | 307 |  | 444 | 456 |
| **66~** | 400 | 400 |  | 304 | 303 |  | 457 | 448 |
| **72-84*** | 400 | 400 |  | 308 | 311 |  | 438 | 456 |
| **Total** | 8778 | 8755 |  | 6690 | 6705 |  | 9309 | 9316 |
|  | | | | | | | | |

* 84 months is not included.
