# Supplementary figures and images for: 20-year trends in prevalence of overweight and obesity among children aged 0-6 in Harbin, China: A multiple cross-sectional study
Source: PLoS One. 2018 Jun 4;13(6):e0198032. doi: 10.1371/journal.pone.0198032 (PMC5986120; doi:10.1371/journal.pone.0198032)

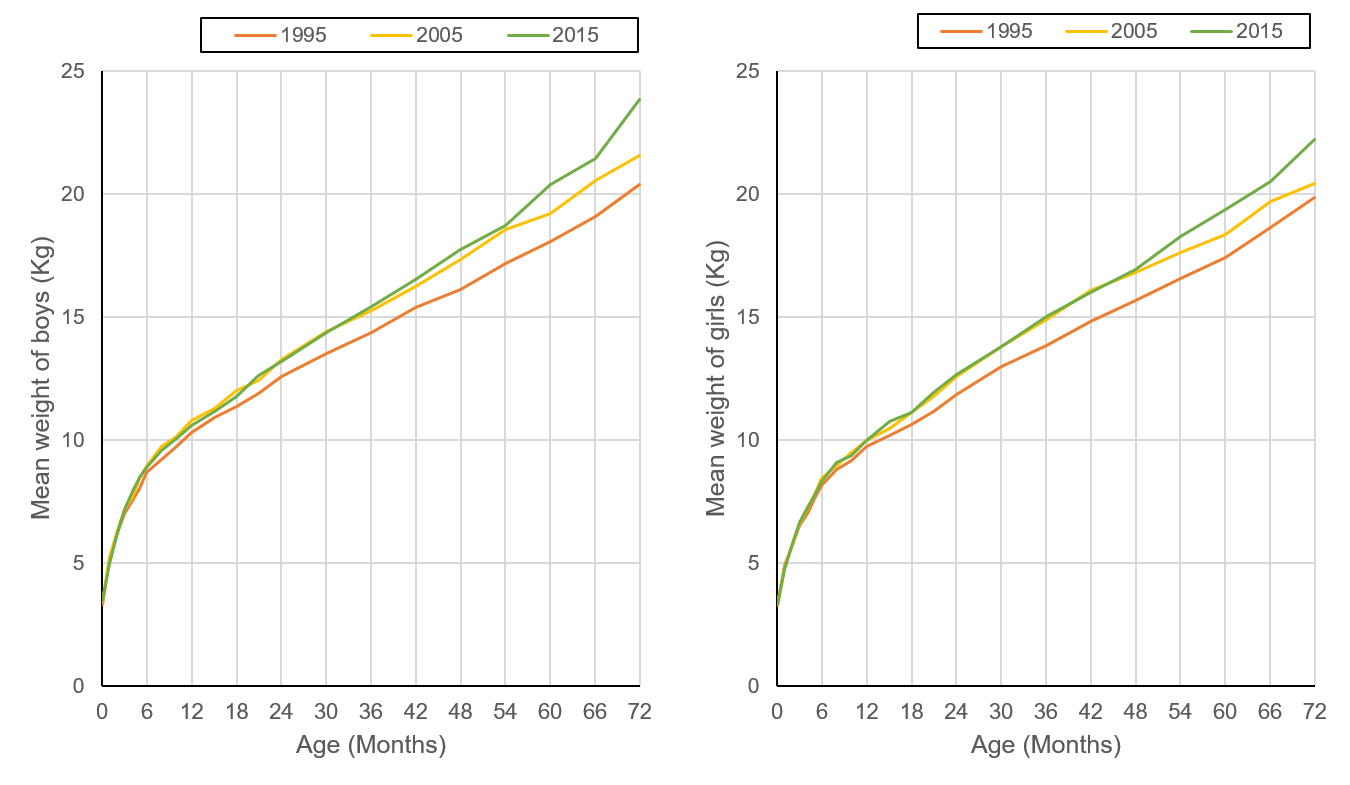

Supplement: S1 Fig — (TIF) [file pone.0198032.s002.tif]

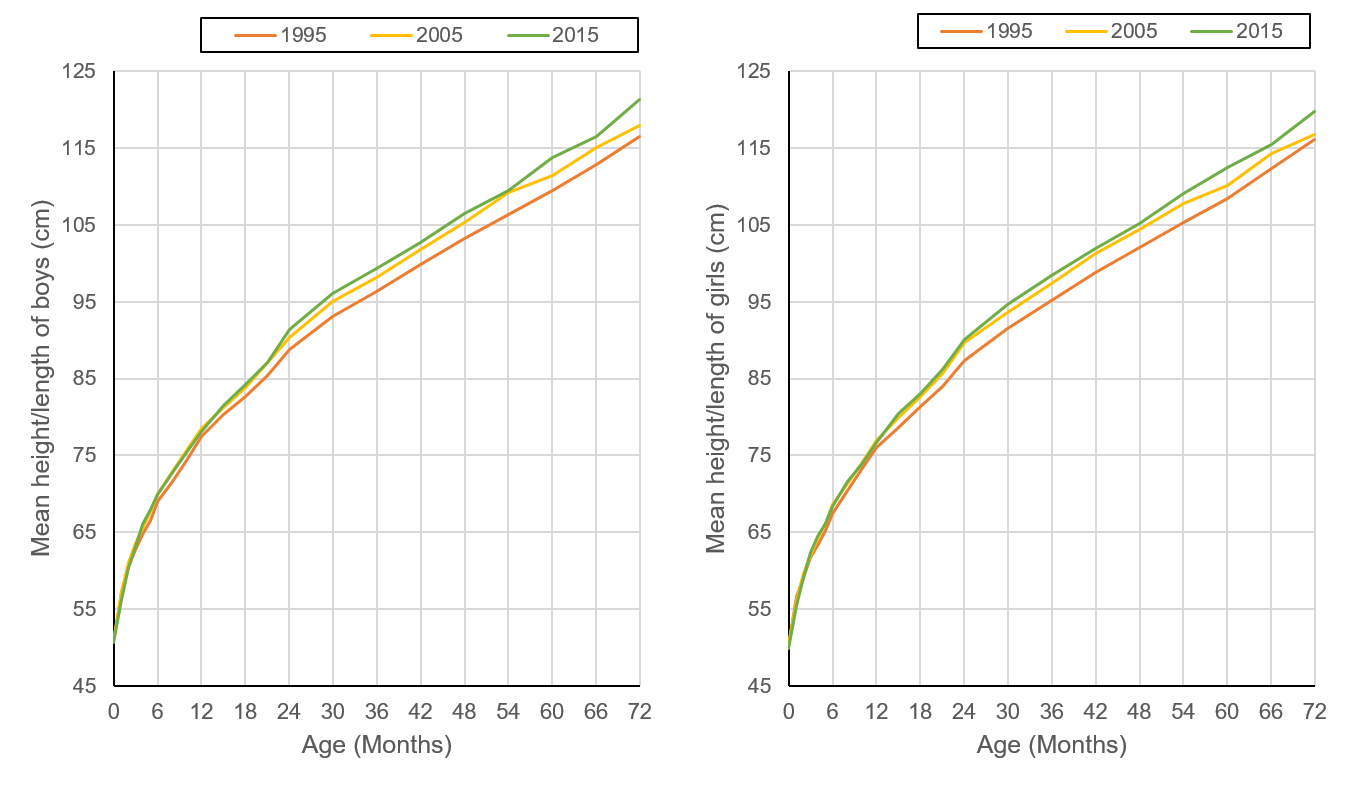

Supplement: S2 Fig — (TIF) [file pone.0198032.s003.tif]

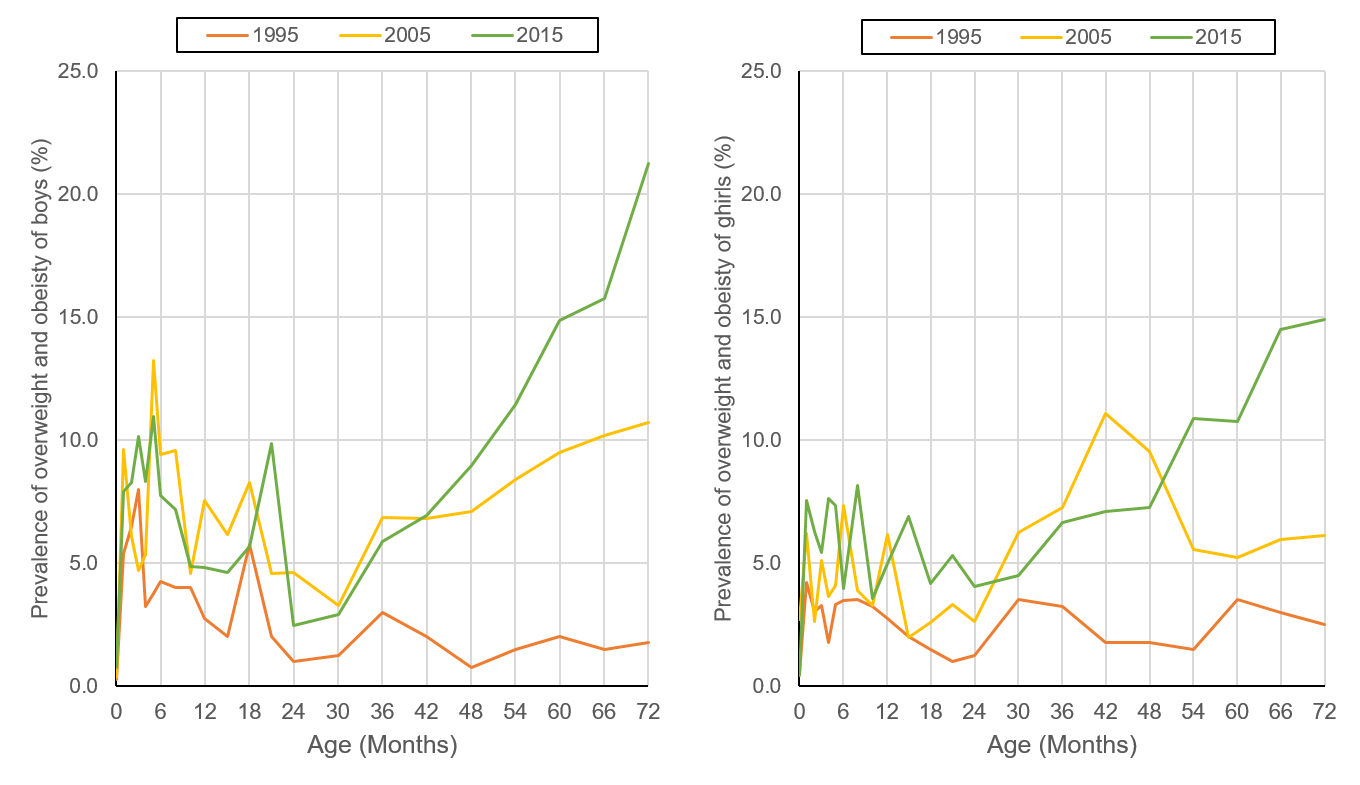

Supplement: S3 Fig — (TIF) [file pone.0198032.s004.tif]
